# Understanding of endo/lysosomal escape of nanomaterials in biomedical application

**DOI:** 10.1002/smo.20240017

**Published:** 2025-03-14

**Authors:** Xin Wang, Haoyu Li, Chen Chen, Zhihui Liang

**Affiliations:** ^1^ School of Chemical Engineering Dalian University of Technology Dalian China; ^2^ CAS Key Laboratory for Biomedical Effects of Nanomaterials and Nanosafety & CAS Center for Excellence in Nanoscience National Center for Nanoscience and Technology of China Beijing China; ^3^ College of Chemical and Biological Engineering Zhejiang University Hangzhou China; ^4^ Shanghai Institute of Materia Medica Chinese Academy of Sciences Shanghai China; ^5^ Shandong Laboratory of Yantai Drug Discovery Bohai Rim Advanced Research Institute for Drug Discovery Yantai China; ^6^ Department of Biomedical Engineering The Chinese University of Hong Kong Shatin Hong Kong

**Keywords:** biomedical application, endo/lysosomal escape, nanomaterials property

## Abstract

Emerging therapies rely on the efficient and specific delivery of targeted agents into the cytosol, such as DNA, siRNA and proteins. Nanoparticles showed great potentials in safe delivery and transportation of the targeted cargoes; however, the entrapment in endosomes and degradation by specific enzymes in the lysosome hindered the bioavailability, cytosolic delivery and subsequent therapeutic efficacy. In this case, the development of methods for efficient and specific delivery of targeted therapeutic agents focuses on overcoming the major challenge of endo/lysosomal escape, which relies on the development of safe and efficient nano‐delivery systems. A deeper mechanistic understanding in the endo/lysosomal escape will guide the development of more efficient nano‐delivery systems. In this review, we summarize various mechanisms by which nanoparticles escape from the endo/lysosome, and showcase the recent progress in dissecting the endo/lysosomal approaches based on nano‐delivery systems. Emphasis will lie on the properties of nanoparticles that govern the endo/lysosomal escape pathway as well as the latest promising applications in vaccine delivery and genetic engineering field.

## Graphical abstract

This review summarized mainstream mechanisms of endo/lysosomal escape and design strategies of endo/lysosomal escapable nanoparticles in view of surface charge, polarity & hydrophobicity, particle morphology, and lysosomal escape agents. The utilization of endo/lysosomal escapable nanoparticles for vaccine delivery and genetic engineering are exemplified, aiming at inspiring the further development of advanced delivery systems for biomedical use.

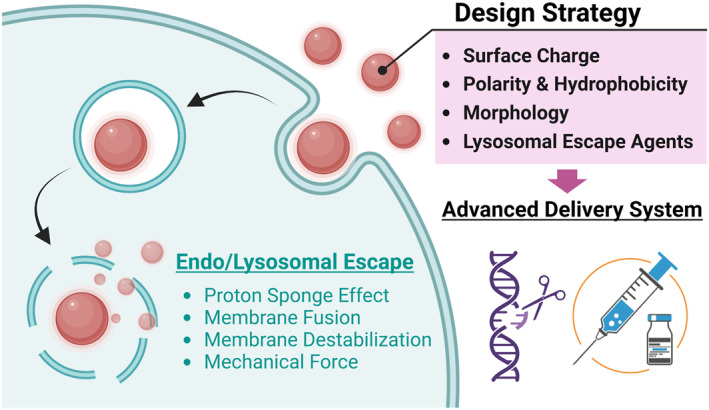

## 1 INTRODUCTION

The endosomal‐lysosomal system consists of a range of organelles responsible for internalizing, recycling, and regulating various cargo molecules, which is an essential part of the endocytosis pathway and plays a vital role in normal cellular function, such as cellular homeostasis, cell growth and proliferation.^[1]^ Endosomes control the recycling of membrane components, thereby modulating fundamental processes in eukaryotic cells, for example, internalization of the nutrient, transduction of deltiological signals, immunity, and adhesion.[^2^, ^3^, ^4^, ^5^] The cargoes endocytosed by different pathways form an endocytic vesicle, subsequently fusing with the early endosome and maturing into the late endosomes.^[6]^ The fusion between the late endosomes and the lysosomes allowed the accumulation of cargoes in lysosomes.^[7]^ To overcome the endo/lysosomal barriers, multiple different types of delivery vehicles have been developed, including liposomes, lipid nanoparticles (LNPs), polymeric nanoparticles, extracellular vesicles, inorganic nanomaterials, *etc.* Nanoparticles, due to their unique physicochemical properties, have been widely used to deliver various therapeutic agents, for example, small molecules, DNA, microRNAs, siRNAs and proteins, *etc*.[^8^, ^9^]

Endocytosis is a necessary physiological process for eukaryotic cells to maintain cellular functions.^[10]^ During the endocytosis, after entering the inner of cells, the cargoes are sorted by the polymorphic and persistent structures of the early endosome, delivered to the late endosome, and finally fused with the lysosome to create hybrid organelles. In this process, pH values gradually decreased from 6.5 to 5.0 (endosomes to lysosomes) with various enzymes (lipases, nucleases, and proteases) produced, resulting in the degradation of cargoes.[^11^, ^12^] Therefore, the overcoming of cargoes endo/lysosomal barriers plays a crucial role in holding bioactive delivery and increasing the therapeutic effect. Over the past decades, to understand the endo/lysosomal escape capabilities of the nanoparticles delivery systems (NDS), multiple mechanisms have been proposed, including proton sponger effect, membrane fusion and mechanical force.^[13]^ For example, polymer nanoparticles have been demonstrated to escape from endo/lysosomes by triggering the proton sponger effect,^[14]^ while LNPs escape by membrane fusion.^[15]^ Overall, NDS showed numerous significant advantages over cargo protection, specific targeting, and precise controlled release. However, the mechanistic understanding on how these nanoparticles contribute to the endo/lysosomal escape remains unclear, which limited the developments and optimal design of promising bionanomaterials for in vitro and in vivo delivery.

In this review, we summarized the recent progress in dissecting the endo/lysosomal escape properties of the nanoparticles and listed the lastest promising biomedical applications, including vaccine delivery and genetic engineering. Currently, most of the published reviews (Table 1) focused on summarizing the endo/lysosomal escape mechanisms that the nanoparticles involved and their biomedical applications, without sorting out the role of the physicochemical properties of the nanopartciels themselves on the endo/lysosomal escape pathway. Here, we capture the intrinsic properties of nanoparticles and showcase why and how these nanoparticles achieved endo/lysosomal escape, providing insights into the rational design of nanomaterials for safe and efficient cytosolic delivery in biomedical applications.

**TABLE 1:** Review articles related to endo/lysosomal topics have been published in recent 5 years.

Title	Focus	Reference
Controlling endosomal escape using nanoparticle composition: Current progress and future perspectives	The relationship between polymer nanoparticles structure and lysosome escape	Cupic et al.^[16]^
The endosomal escape of nanoparticles: Toward more efficient cellular delivery	Mechanism of lysosome escape, emerging techniques to improve lysosome escape	Smith et al.^[17]^
Strategies in the design of endosomolytic agents for facilitating endosomal escape in nanoparticles	Compare endosomal escape mechanisms of viruses with different nanoparticles	Ahmad et al.^[18]^
Tailoring iron oxide nanoparticles for efficient cellular internalization and endosomal escape	Role of iron oxide nanoparticles surface modification alternatives for lysosome escape	Gensini et al.^[19]^
Escaping the endosome: Assessing cellular trafficking mechanisms of non‐viral vehicles	Experimental techniques for assessing the extent of endosomal escape of a variety of non‐viral vehicles	Xu et al.^[20]^
Strategy for cytoplasmic delivery using inorganic particles	Strategies in the design of inorganic nanoparticle‐based cellular delivery	Xu^[21]^
Nanomaterials respond to lysosomal function for tumor treatment	Role of direct destruction of lysosomes or lysosomal escape in tumor treatment	Tian et al.^[22]^
Endosomal escape of bioactives deployed via nanocarriers: Insights into the design of polymeric micelles	Mechanism of lysosome escape, polymeric micelles used for endosomal escape	Butt et al.^[23]^
Endosomal escape for cell‐targeted proteins. Going out after going in	Mechanisms intended to evade lysosomal degradation of proteins	Duran et al.^[24]^

## 2 MECHANISMS GOVERN ENDO/LYSOSOMAL ESCAPE

**FIGURE 1:**
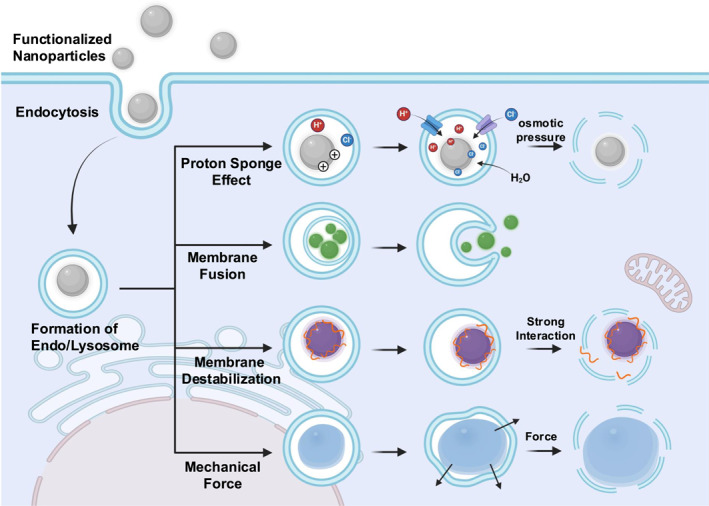
Representative mechanisms for endo/lysosomal escape. Created with BioRender.com.

Nanoparticle uptake via endocytosis involves the following four main processes: internalization into endocytosed vesicles, fusion into early endosomes, maturation in late endosomes, and accumulation in lysosomes (IFMA Cascade).^[17]^ Particles are expected to accomplish lysosomal escape during endosome‐lysosome formation, otherwise they will be degraded by lysosomes.^[25]^ In this section, we elucidate various influential mechanisms of endosomal escape, including proton sponge effect, membrane fusion, membrane destabilization and mechanical force‐derived endosomal escape (Figure 1).

### 2.1 Proton sponge effect

“Proton sponge” effect was proposed by Behr et al. in 1997.^[26]^ It is often described as the most likely mechanism of the lysosomal escape: during the acidification process of endosomes, the delivery carriers with buffering capacity inhibit the drop of pH and cause cells to continue pumping protons into endosomes to achieve the desired pH value. To balance the charge inside the vesicles, Cl^−^ and H_2_O are transported into the endosomes, increasing the pressure of endosomes and ultimately lysing the endosomes.

Cationic polymers are common carriers that help cargos escape from endo/lysosomes through the proton sponge effect due to their excellent buffering capacity near physiological pH. Straight‐ and branched‐chain polyethyleneimine (PEI) is one of the most widely studied polycations in cytoplasmic delivery of nucleic acids, due to its high buffering capacity at endo/lysosomal pH and correspondingly high endo/lysosomal escape efficiency.^[27]^ The positive charge density of cationic polymers is closely related to the degree of polymerization. Research by Jokerst et al. demonstrated that branched PEI with high molecular weight (25 kDa) exhibited a much higher positive surface charge density than that with low molecular weight (800 Da), which may be attributed to the strong protonation of rich amine groups.^[28]^ The positive surface charge density of low‐molecular‐weight branched PEI (ow‐molecular‐weight branched PEI (lwPEI)) was enhanced when lwPEI self‐assembled “high molecular weight” nanoparticles, resulting in a significant enhancement in the lysosome rupturing capability. It is worth noting that compared to PEI, the protons absorb ability of polylysine (PLL) is weak, which could be enhanced by the modification of polymer materials with histidine. Langer et al.^[29]^ complexed plasmid DNA ( plasmid DNA) loaded PLL with gluconic acid modified polyhistidine, which showed a significant improvement in the buffering capacity and transfection efficiency of the complex. Taken together, from the perspective of the proton sponge effect, the introduction of amino groups effectively promotes the lysosome escape by improving the buffering capacity of the polymers.

### 2.2 Membrane fusion

Membrane fusion is a vital process with broad varieties in facilitating transmembrane transport of molecules between cells or subcellular structure in living organs.^[30]^ During this process, vesicle‐loaded cargos are able to escape from the endo/lysosome and release into the cytoplasm by forming direct channels between the vesicle structure and the cytoplasm.^[25]^ This particular mechanism is widely involved when assisting the viral infection process, as endo/lysosomal acidification leads to conformational changes of the viral envelope, which further allows the endo/lysosomal membrane penetration of the viral structure and the subsequent release of viral genome.[^31^, ^32^] Various synthetic systems have been developed to mimic this endo/lysosomal‐activable membrane fusion event as a bottom‐up delivery approach. Basically, the key to realize the lysosomal escape through membrane fusion lies in the rational design of the composition and structure of the outer membrane of nanoparticles. A common strategy is to introduce membrane fusion agents into vesicle‐like delivery systems, referring to natural viral composition. Various fusogenic peptides, such as HA2 (GLFGAIAGFIENGWEGMIDG), KALA (WEAKLAKALAKALAKHLAKALAKALKA), GALA (WEAALAEALAEALAEHLAEALAEALEALAA),[^33^, ^34^, ^35^, ^36^] have been used to direct membrane fusion upon a specific endo/lysosomal trigger, hence leading to a more efficient endosomal escape. These fusion peptides share a similar membrane fusion mechanism, which is that they form a fusogenic α‐helical structure under certain conditions (HA2 and GALA transform to α‐helical conformation at endo/lysosomal acidic environment, while KALA exist a helical structure at both physiological and acidic pH values).^[37]^ In addition, the structure of nanoparticles is critical for the endo/lysosomal escape. LNPs are regarded as the most promising carriers of nucleic acids. There are three main categories of reported LNP‐nucleic acid nanostructures: L, H_II_, and Q_II_. Previously, periodic bicontinuous cubic membrane interiors in LNPs have been demonstrated to lower the elastic cost of inducing membrane fusion.^[38]^ Cubic and inverse hexagonal structured LNP‐RNA complexes are able to fuse with endosomal membranes, leading to higher endosomal escape efficiency.[^38^, ^39^] Interestingly, the shape of lipoplexes was demonstrated to change from lamellar to inverted H_II_ hexagonal phage when the ratio of 2‐dioleoyl‐*sn*‐glycero‐3‐phosphoethanolamine (DOPE) increased, which induces the fusion of the outmost liposomal membrane with endosomal membranes.[^40^, ^41^, ^42^]

### 2.3 Membrane destabilization

Endo/lysosomal escape through membrane destabilization in nanoparticle‐mediated delivery involves intricate interactions between the nanoparticles and the bio‐membrane.^[43]^ Unlike the proton sponge effect, this process lays emphasis on the direct interaction between the bio‐material interface rather than damage to the endo/lysosomal membrane indirectly caused by changes in surrounding components.^[44]^ The surface charge, functional groups, hydrophobicity or hydrophilicity, and even morphological characteristics of nanoparticles significantly impact their interactions with biological membranes.[^11^, ^45^]

Both membrane destabilization and membrane fusion mechanisms involve direct interaction between nanoparticles and endo/lysosomal membranes. It should be noted that escape through membrane destabilization rather than membrane fusion causes over‐exposure of lysosomal internal environment to the cytoplasm. For instance, GALA peptide undergoes a structural transformation in the acidic pH of endosomes and will easily insert into the lipid bilayer.^[46]^ It embeds in two parallel membranes (vesicle‐like particles and endo/lysosome), thus leading to a close contact of the two membranes at the GALA site, which is the start point of membrane fusion.[^47^, ^48^] However, in non‐vesicle systems, GALA was reported to insert into the endo/lysosomal membrane to form pores directly, resulting in the escape of cargos from the endo/lysosome through membrane destabilization.[^49^, ^50^]

For complex delivery systems, the methods by which they influence lysosomal bioactivity extend beyond those effects merely attributed to surface charges, including the proton sponge effect. This intricate interplay suggests a multifaceted approach to enhance the endosomal escape of nanoparticles beyond the simplistic view of charge‐dependent mechanisms. For instance, the mechanism of lysosomal escape mediated by cationic liposomes has been debated in recent studies. Some researchers proposed that cationic liposomes facilitate escape by disrupting endo/lysosomes via the proton sponge effect,[^51^, ^52^] while others suggested that the positively charged lipids interact with the negatively charged endosomal membranes, thus facilitating the membrane fusion and destabilization.[^38^, ^53^] Liu et al. formulated nanoparticles with lipid molecules containing amphipathic heads (tertiary amine group), one phosphate group and three hydrophobic tails (multiple alkyl chains).^[54]^ Upon entry into acidic endosomes/lysosomes, the tertiary amine undergoes protonation, inducing the integration of the lipid molecules into the lysosomal membrane, catalyzing a phase transition of membrane towards a hexagonal configuration, thus facilitating effective lysosomal escape and release of nucleic acid cargos inside.

In addition to the proton sponge effect, polymer‐mediated direct membrane destabilization was involved during the lysosome escape, since the insertion of free cationic polymer chains into the endosomal membrane enhances its permeability.^[55]^ Polymer‐mediated direct membrane destabilization has been demonstrated to be involved due to the insertion of free cationic polymer chains into the endosomal membrane to enhance its permeability. Previours studies showed that PEI aggregates adhere to the inner lysosomal membrane, leading to partial membrane disruptions.[^56^, ^57^] The positively charged amino groups within PEI exhibit a propensity to repel each other, which leads to an expansion of the volume of the polycations associated with the polyplexes in acidic conditions, a phenomenon known as the umbrella effect.[^58^, ^59^] Consequently, this may directly disrupt the membrane. A direct interaction between the polymer and the membrane, driven by charge neutralization, induces local membrane destabilization or the formation of nanoscale pores, facilitating the release of the polyplexes into the cytoplasm.^[60]^ This approach primarily aims at introducing pores in the membrane rather than completely disrupting it, which reduces the release of enzymes from lysosomes, decreasing the subsequent impact on mitochondrial function and ensuring the safety of this lysosomal escape mode.^[60]^

### 2.4 Mechanical force

Nanoparticles directly cause physical damage to the lysosomal membrane through mechanical forces, mainly involving dynamic morphological changes or gas release from the particles inside the lysosomes. Recently, studies have developed “nanomotors” that vibrate within lysosomes, thereby damaging the lysosomes owing to their motility.[^61^, ^62^] Chen et al.^[61]^ have constructed a nitric oxide (NO)‐driven nanomotor that can move in the tumor microenvironment to deliver chemotherapeutic drug docetaxel (DTX) and immune checkpoint inhibitor anti‐PD‐1 (aPD1). The study shows that plenty of cargos loaded on nanomotors, rather than those loaded on vehicles without motion ability, appear outside the lysosome, which illustrates that motility properties (mechanical force) contribute a lot to good lysosomal escape performance.

## 3 DESIGN OF ENDO/LYSOSOMAL ESCAPABLE NANOMATERIALS

In numerous pharmacological studies (including vaccines, genetic drugs, anti‐inflammatory drugs, and anti‐cancer drug delivery), a common challenge is the entrapment of drugs within lysosomes, leading to a significant reduction in therapeutic efficacy. In this section, we focus on the physicochemical properties of nanomaterials themselves, including surface charge, hydrophobicity, and morphological characteristics, to elucidate how these properties play roles in the interaction with the endo/lysosomal systems.

### 3.1 Surface charge

Regulation of surface electric properties contributed to enhance the endo/lysosomal escape efficiency of nanomaterials. Currently, lysosome escape induced by the surface charge of nanomaterials mainly occurs through two mechanisms, including proton sponge effect and/or positively charged surfaces induced lysosomal membrane fusion/instability. Typically, nanoparticles are functionalized cationic groups on their surface, or encapsulated with cationic lipids or polymers, to endow the materials with a positively charged surface within the lysosomal environment.

**FIGURE 2:**
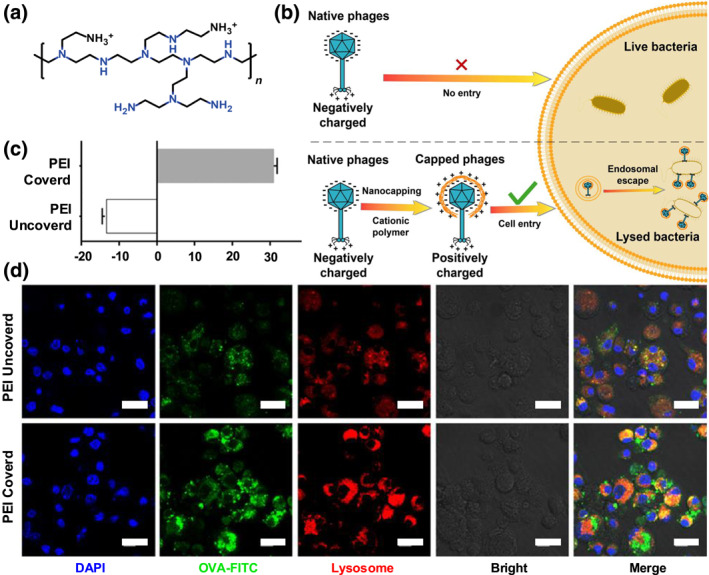
(a) Chemical structure of polyethyleneimine (PEI). (b) Schematic illustration of cationic nanocapping‐mediated charge reversal of phages, facilitating their cellular entry and subsequent endosomal escape. Reproduced with permission.^[63]^ Copyright © 2022, American Association for the Advancement of Science. (c) Surface zeta potential of the nanovaccine before and after PEI modification. (d) Confocal images of DC2.4 cells after co‐incubation with 15.625 μg/ml of PEI coated/uncoated nanovaccine (scale bar = 20 μm). Reproduced with permission.^[64]^ Copyright © 2019, American Chemical Society.

Polyethyleneimine is a classic cationic polymer for cytosolic delivery (Figure 2a). The proton sponge effect is predicated on extensive buffering capacity of PEI, attributable to its primary, secondary, and tertiary amine groups. These groups, upon protonation, are able to elevate the pH within the endo/lysosomal compartment.^[58]^ Forrest et al.^[65]^ generated PEI derivatives through acetylation of primary amines, resulting in a decrease in the buffering capacity of the polymer. Compared with unmodified PEI, the transfection efficiency of the modified PEI group increased 21 times. PEI‐modified nanoparticles are excellent non‐viral nucleic acid delivery carriers, widely used in the delivery of small interfering RNA, nucleic acid drugs, and bacteriophages for gene editing or immunotherapy. For example, Meng et al.^[63]^ delivered bacteriophages into cells to inhibit the invasion of intracellular pathogens. To enhance the bioavailability of the bacteriophages, PEI was used to coat the negatively charged heads of bacteriophages by electrostatic interactions. This capping of bacteriophages not only facilitated their entry into the intestinal epithelial cells but also enabled their escape from endosomes through the proton sponge effect, thereby lysing the host cells carrying pathogens (Figure 2b). In addition, PEI coating on the nanovaccines facilitated the cellular uptake by immune cells and further lysosomal escape of nucleic acid/protein antigens, promoted antigen cross‐presentation, thus enhancing the antigen‐specific CD8^+^ T cell response. Gu et al.^[64]^ developed ovalbumin (OVA)‐loaded poly(lactic‐co‐glycolic acid) (PLGA) nanoparticles coated with PEI, which exhibit positive charges (Figure 2c). Confocal images showed that compared to the uncoated group, FITC labeled OVA was less colocalized with lysosomes in PEI coated PLGA group (Figure 2d), indicating the successful endo/lysosomal escape, leading to the enhanced antigen‐specific CD8^+^ T cytotoxic cell activation and responses.

Cationic liposomes and LNPs, are excellent carriers of nucleic acids and proteins. They are usually positively charged under endo/lysosomal acidic conditions, thereby effectively inducing lysosomal escape to enhance the bioavailability of internal cargoes. Shi et al.^[66]^ developed cationic liposomes composed of 1,2‐dioleoyl‐3‐trimethylammoniumpropane (DOTAP), DOPE, and 1,2‐distearoyl‐*sn*‐glycero‐3‐phosphorylethanolamine (DSPE)‐poly(ethylene glycol) (PEG) to encapsulate SARS‐CoV‐2 receptor binding domain mRNA to achieve lysosomal escape and enhance the efficiency of mRNA cytosolic delivery. The utilization of cationic lipids in pharmaceutical formulations embodies a paradox, as their incorporation, while facilitating lysosomal membrane destabilization, raises significant safety concerns due to the absence of targeted induction of membrane destabilization. In light of the drawbacks associated with the indiscriminate membrane fusion caused by these excipients, recent years have witnessed a surge in the exploration of pH‐sensitive liposomes.^[67]^ These liposomes are distinguished by their ability to rapidly discharge their contents in response to the progressively acidic environment of the endosome, thereby offering a promising avenue to mitigate the off‐target effects of drug therapies and enhance the biocompatibility of lipid‐based delivery systems.^[68]^

However, positively charged surfaces may induce cytotoxicity and reduce particle circulation time in vivo. To mitigate this challenge, several innovative carrier designs have been proposed, incorporating charge‐conversion strategies. These approaches enable built‐in functionalities to switch from anionic to cationic in response to environmental stimuli, such as pH, redox, reactive oxygen species (ROS), enzyme, light or temperature, allowing the carrier to overcome critical barriers in therapeutic delivery, achieve local targeted drug release, and facilitate endo/lysosomal escape.[^69^, ^70^, ^71^, ^72^] For example, Shi et al.^[73]^ modified the surface of cationic nanocarriers with a layer of hyaluronic acid, enabling the nanoparticles to passively target and infiltrate tumor sites. Upon exposure to hyaluronidase, the surface charge of nanoparticles switches from negative to positive, enhancing tumor cell uptake and facilitating drug cytosolic delivery through lysosomal escape via the sponge effect.

### 3.2 Polarity and hydrophobicity

**FIGURE 3:**
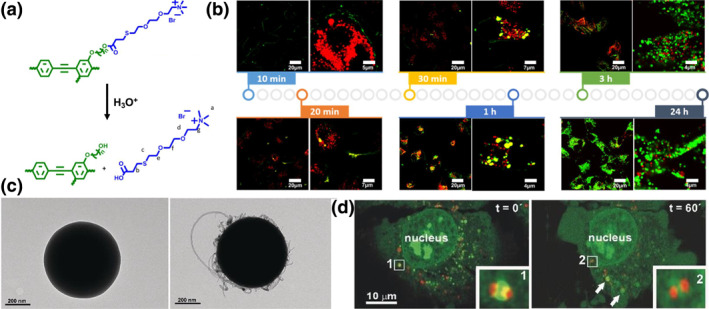
(a) Chemical structure and hydrolysis process of the polarity‐switchable polymer. (b) Intracellular co‐localization image of PPE‐A (Green) and lysosome (Red) of A549 cells after incubation with 2.5 μM PPE‐A for different times. Reproduced with permission.^[77]^ Copyright © 2017, American Chemical Society. (c) Transmission electron microscopy (TEM) images of SiO_2_ particles (left) and SiO_2_ particles coated with carbon nanotubes (CNTs) (right). (d) Live‐cell confocal images of CNTs‐coated silica nanoparticles in HeLa cells. Particle polarization‐induced endo/lysosomal membrane permeabilization, leading to the lysosomal escape of the nanoparticles (Red). Reproduced with permission.^[78]^ Copyright © 2017 Wiley‐VCH.

Nature has developed a mature system for species to evade the arrest by lysosomes based on polarity and hydrophobicity. Some viruses and bacteria escape from endosomes by peptides such as cationic amphiphilic peptides (amphiphilic peptides), which generate and stabilize pores in the lipid membrane.^[74]^ Influenza viruses fuse into the endosome membrane through hemagglutinin, which could convert from an anionic hydrophilic coil to a hydrophobic α‐helical structure at acidic environment.^[75]^ It was reported that nanoparticles with higher hydrophobicity are prone to permeate membranes and induce stronger endo/lysosomal escape or rupture.^[76]^ Zhou et al.^[77]^ designed a rigid‐backboned conjugated polymer with pH‐sensitive cleavable water‐soluble side chains (PPE‐A), which undergoes a polarity switch triggered by the acidic environment within lysosomes (Figure 3a). Upon internalization into the lysosome, these side chains are cleaved, resulting in a transformation of the polymer from a hydrophilic to a highly hydrophobic entity. As a consequence, the polymer loses its solubility in water, precipitates, and expells from the lysosome due to its hydrophobic nature, thereby achieving cytoplasmic delivery of inside cargoes (Figure 3b). Interestingly, inspired by natural structures like virus capsids, nanoparticles are able to be engineered with specific surface patterns to enhance their interaction with biological interfaces. Rodríguez et al. explored a novel surface coating made from adsorbed carbon nanotubes (CNTs).^[78]^ This coating grants the particles virus‐like properties (Figure 3c), including receptor‐mediated endocytosis, endo/lysosomal escape, and release profile while preserving cell viability (Figure 3d). The hydrophobic regions of CNT nanotubes will be exposed in the lysosomes to enhance the affinity of the nanoparticles to lipid membranes, triggering membrane penetration, perforation, permeabilization, thus enhancing the delivery efficacy of various therapeutic agents, including proteins and nucleic acids.

### 3.3 Morphology

**FIGURE 4:**
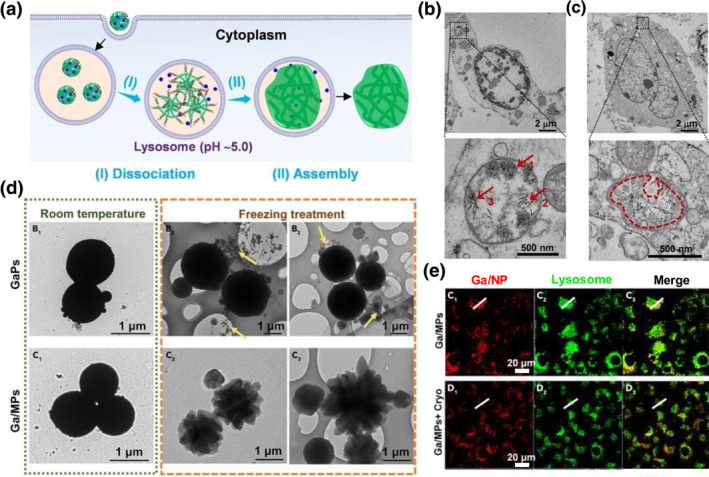
(a) Scheme illustration of topological transformation from nanoparticle to organelle‐like hydrogel architecture in response to lysosomal acidic micro‐environment. The nanoparticles swell and destroy the lysosomal membrane by mechanical force, leading to lysosomal escape. (b)–(c) Bio‐transmission electron microscopy (TEM) images of U87 MG cells treated with 5 μg/mL (b) immutable nanoparticles and (c) expandable nanoparticles. Reproduced with permission.^[79]^ Copyright © 2020 Wiley‐VCH. (d) TEM images of immutable/expandable particles under room temperature and freezing treatment. Ga fragments are indicated by the yellow arrows. (e) Intracellular co‐localization images of expandable particles with lysosomes under room temperature and freezing treatment. Reproduced with permission.^[80]^ Copyright © 2021 Elsevier.

In comparison to optimizing the physicochemical properties of nanoparticles to promote the endo/lysosomal membrane functionality, utilizing the unique morphology of nanomaterials, or their dynamic morphological changes to rupture lysosomal membrane through mechanical forces, offers a more direct and reliable lysosomal escape strategy. Some particles are designed to swell selectively after entering endo/lysosomes in response to the acidic environment, thereby directly escaping from the lysosome. Guo et al.^[79]^ developed a double‐stranded DNA (dsDNA) molecule with two cytosine‐rich single‐stranded sticky ends. This dsDNA spontaneously aggregates to form nanoparticles in the presence of Mg^2+^. Interestingly, under acidic conditions, these nanoparticles disassociate and reform a hydrogel‐like structure through phase separation. In this process, the particles swell and further rupture the lysosome membrane to facilitate cytoplasmic delivery (Figure 4a). Bio‐transmission electron microscopy image shows that immutable nanoparticles (red arrows) are trapped in lysosomes, while expandable nanoparticles form organelle‐like hydrogel by topological transformation (Figure 4b–c). The dynamic morphological changes that damage the lysosomal membrane are not limited to the expansion of the nanoparticle volume. Wang et al. used cell membranes to encapsulate ionic liquids, which undergo drastic shape transformations within lysosomes, forming urchin‐like particles that pierce through the liposome membrane, thereby directly rupturing the lysosome (Figure 4d–e).^[80]^ Taken together, by manipulating the interactions within the internal structure of nanoparticles and incorporating responsive functionalities, nanomaterials are able to undergo morphological transformations within lysosomes, to achieve lysosomal membrane damage and facilitate their release from lysosomes.

### 3.4 Lysosomal escape agents

**FIGURE 5:**
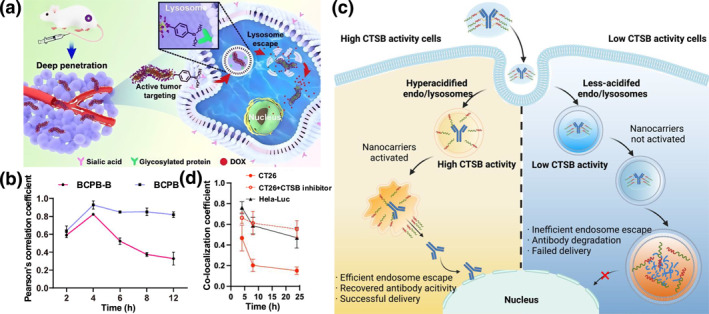
(a) Schematic illustration of PBA‐tiggered lysosomal escape of PBA‐modified cylindrical polymer brushes (BCPB‐B). (b) Time‐dependent evolution of the Pearson's correlation coefficients between cylindrical polymer brushes (CPB) and lysosomes in CT26 cells following incubation with 10 μg/ml of BCPB‐B and BCPB (CPB without phenylboronic acid [PBA] modification). Reproduced with permission.^[85]^ Copyright © 2021 American Chemical Society. (c) Schematic illustration of a CTSB‐sensitive system facilitating cell‐specific intracellular antibody delivery via a selective endosomal escape pathway. (d) Time‐dependent evolution of the co‐localization coefficients between antibodies and lysosomes in CT26 cells, with or without CTSB inhibitor treatment. Reproduced with permission.^[86]^ Copyright © 2024 Wiley‐VCH.

Instead of modulating the surface properties of nanomaterials, some nanoparticles are modified with or carried with lysosomal escape agents to facilitate the penetration or disruption of lysosome membranes, such as membrane fusion peptides, cell‐penetrating peptides, and endo/lysosome‐targeting molecules.[^81^, ^82^, ^83^, ^84^] For example, Wang et al.^[85]^ modified cylindrical polymer brushes (CPB) with phenylboronic acid ( phenylboronic acid (PBA)) groups to facilitate the lysosome escape and build a cytoplasm‐delivery platform for anti‐tumor drugs, PBA‐modified cylindrical polymer brushes. They speculated that PBA specifically interacts with lysosomal membrane proteins and hot shock proteins, thus facilitating the lysosome escape (Figure 5a–b). A proper modification will benefit site‐specific endo/lysosomal escape. Chen et al.^[86]^ modified a novel antibody delivery system with cathepsin B (CTSB)‐sensitive groups. As cancer cells show higher CTSB activity than normal cells, this delivery system will allow selective endo/lysosomal escape of antibodies towards cancer cells, hopefully avoiding harmful side effects (Figure 5c–d).

Moreover, a series of delivery carriers are capable of releasing lysosomal escape agents in response to the endo/lysosomal environment, thereby enhancing the lysosomal escape of inside cargos. For instance, as a pH sensitive inorganic nanomaterial, calcium phosphate (CaP) is able to produce calcium ions in the acidic micro‐environment,^[87]^ increase the osmotic pressure and generate the proton sponge effect to enhance endosomal escape.^[88]^ Recently, Zhang et al.^[89]^ prepared tumor‐targeting peptide (RGD)‐modified DSPE‐PEG micelles to load the mitochondrial‐targeting PTT dye (IR780) and Ca^2+^ efflux pump inhibitor curcumin (^R^M_(I+C)_). Calcium phosphate mineralizes on the PEG layer of the micelle system, forming the final nanodevice ^R^M_(I+C)_@CaP_(P)_. After cellular internalization, CaP generates a large amount of Ca^2+^ to facilitate lysosomal escape, thereby achieving the silencing of HSP70 mRNA. Manganese dioxide nanoparticles are capable of catalyzing the decomposition of H_2_O_2_ to O_2_, thereby facilitating the rupture of lysosomes by gas generation. Ye et al. developed a nanomotor capsulated Manganese dioxide antennas, which convert ROS into oxygen bubbles to serve as a power source for the nanomotor activation.^[90]^ The rapid oscillations of the nanoparticles, along with the generated oxygen, inflict the mechanical damage on the lysosomal membrane, thus aiding in the lysosomal escape of particles. In addition, certain inorganic carbonates were designed to serve as viable carriers for lysosomal escape, for example, calcium carbonate (CaCO_3_) nanoparticles. Yin et al.^[91]^ mixed RNase A, glucose oxidase (GOD), Tannic acid, and Mn^2+^ in one pot to attain RG@MT, and coated nanoparticles with CaCO_3_ by biomineralization. The CaCO_3_ layer degrades in response to the lysosomal acidic environment, produces CO_2_ bubbles and thus leads to lysosome bursting. This process ensures the co‐delivery of protein drugs, RNase A, and GOD, as well as their escape from lysosomes and release into the cytoplasm.

## 4 APPLICATIONS

Nanoparticles provide an appropriate vehicle for transporting internal drugs through various biological barriers to reach target cells. However, some of them fail to exert their desired therapeutic effect due to the entrapped nanoparticles in lysosomes after entering the cells. In this section, we primarily focus on the application of nanoparticle lysosomal escape strategies in vaccine delivery, tumor treatment, and gene editing, to understand how endo/lysosomal escape improves the therapeutic effects of modern nanoparticles.

### 4.1 Vaccine delivery

Recent outbreaks of various infectious diseases have stimulated the rapid advance of nanovaccine technology. During the development of nanovaccines, it has been observed that some vaccines do not achieve the expected immunogenic effects due to the entrapment of vaccine components within lysosomes after endocytosis, leading to enzymatic degradation and failure to reach the site of action, thereby significantly reducing the bioavailability of the materials.

**FIGURE 6:**
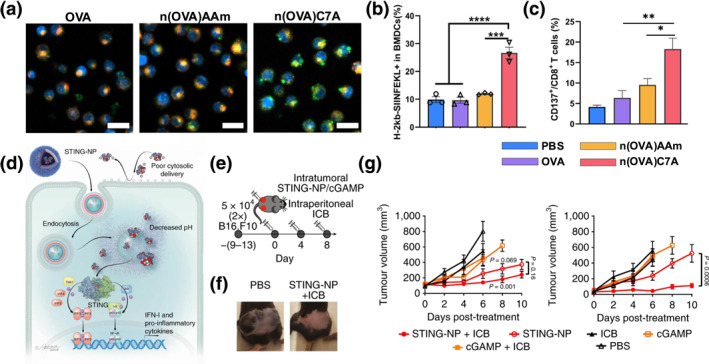
(a) Intracellular co‐localization image of OVA (Green) and lysosome (Red) in DC2.4 cells after incubation with 10 μg/ml n(OVA)C7A nanovaccine and control groups for 24 h (scale bar = 40 μm). (b) Flow cytometry analysis of H‐2K^b^/SIINFEKL^+^cell population in bone marrow‐derived dendritic cells (BMDCs) after incubation with nanovaccines. (c) Percentage of CD8^+^ T cells activated in lymph nodes after vaccine treatment. Reproduced with permission.^[94]^ Copyrights © 2024 Elsevier. (d) Schematic illustration of cGAMP cytosolic delivery by STING‐NPs via pH‐responsive endosomal escape process. (e) Treatment scheme for mice treated intravenously with vaccine formulations and intraperitoneally with immune checkpoint blockade (ICB). (f) Representative images of tumors 8 days after initial treatment. (g) Tumor growth curves following intravenous injection of the formulations. Reproduced with permission.^[95]^ Copyright © 2019 Springer Nature.

Most antigens, including proteins, peptides, and mRNA, require processing or translation within the cytoplasm of antigen‐presenting cells prior to presentation of antigen information to T cells. In a recent study,^[92]^ black phosphorus nanosheets (BP) delivery system was developed to neutralize hydrogen ions within lysosomes, thereby increasing phosphate production and enhancing the lysosomal escape of inside cargoes. This alteration in the lysosomal environment causes the swelling and rupture of the lysosomes, facilitating the release of enclosed mRNA into the cytoplasm. For protein and peptide vaccines, lysosomal escape is usually necessary to prevent excessive degradation of antigen components. Lysosomal escapable delivery systems enable the cytoplasmic delivery efficiency of internal protein/peptide antigens, and subsequently promote antigen cross‐presentation and antigen‐specific CD8^+^T cell activation.^[93]^ Zhao et al. designed a polymeric nanovaccine, n(OVA)C7A, by covering OVA with pH‐responsive 2‐(hexamethyleneimino) ethyl methacrylate (C7A‐MA), acrylamide (AAm), and the biodegradable crosslinker N,N′‐Bis(acryloyl) cystamine.^[94]^ Compared to the control group, n(OVA)C7A exhibited an enhanced endosomal escape of the model antigen OVA (Figure 6a). This efficient endosomal escape further contributed to the increased the expression level of the MHC‐I‐presented peptide SIINFEKL and enhanced activation of CD8^+^ T cells (Figure 6b–c).

In addition to antigens, vaccine adjuvants are also needed to avoid premature degradation during vaccine delivery through lysosomal escape, especially in inactivated and recombinant subunit vaccines.^[93^ ^,^ ^96^, ^97^, ^98^ Most vaccine adjuvants work in the cytoplasm, such as the stimulator of interferon genes agonist 2′3′‐cyclic GMP‐AMP (2,3‐cGAMP), which easily loses its biological activity in the lysosomes.^[99]^ By designing nanocarriers capable of lysosomal escape, both antigens and 2,3‐cGAMP are delivered to the cytoplasm simultaneously.^[95]^ Shae et al. encapsulated cGAMP within polymersomes as nanovaccines (STING‐NP) to alleviate tumor growth in a poorly immunogenic B16F10 melanoma model, significantly stimulating IFN‐I responses in monocytes, macrophages, and melanoma cells, enhancing the subsequent tumor growth inhibition effects (Figure 6d–e).^[95]^ This encapsulation approach exploits the ability of the nanoparticles to escape lysosomal degradation, thereby ensuring the effective delivery and activation of cGAMP, which plays a crucial role in mediating immune responses against tumor cells. Further optimization and understanding of these nanodelivery systems will significantly improve therapeutic outcomes in clinical treatments by enhancing the immunogenicity and efficacy of intracellularly active pharmaceutical compounds.

### 4.2 Genetic engineering

Disease treatment using gene‐based therapies has gained significant attention over the past decades.^[100]^ The fundamental idea of gene therapy is to alter or modify defective/missing gene sequences to cure inherited diseases.^[101]^ Therapies based on genetic engineering have broad application prospects; however, transfecting cells with oligonucleotides is inefficient due to the low efficiency of cellular degradation and cellular internalization.^[101]^ Gene delivery using non‐viral vectors, including lipoplexes and polyplexes, is still of limited efficiency.^[102]^ Apart from being confronted with various extracellular hurdles, additional barriers arise for nanocarriers when they encounter the target cells. Effective internalization and subsequent release of their cargo required translocation across endosomal and/or nuclear membranes. Therefore, efficient nucleic acid delivery systems are needed that ideally escape from endosomes and promote intracellular delivery. In the field of gene delivery, nanoparticles have been translated into the clinic as a promising platform. It has been widely studied and applied in improving the efficiency of gene delivery by inducing lysosomal escape.

Genome therapies based on DNA have been extensively studied due to their great potential in treating cancer, cardiovascular, infectious and monogenic diseases. With the advent of new gene engineering technologies, therapeutic DNA sequences of interest are often combined with zinc‐finger nucleases sequence and transcription activator‐like effector or clustered regularly interspaced short palindromic repeats (CRISPR)/Cas9. This is a complex molecular machinery that once enters the cell nucleus, acts by correcting, adding, or knocking down the DNA corrupted gene of interest. These steps eventually modify the protein expression, production and stability. The CRISPR/Cas9 system is a promising approach for gene editing in gene therapy.^[103]^

Polymeric DNA vectors have the advantage of broad functionality, which enhances their potential to resist degradation in the body. At the same time, based on the design of monomers that promote lysosomal escape, polymerer can significantly improve the delivery efficiency of DNA. For example, the two most commonly used polymeric vectors, PLL and PEI functionalized with PEG, have been clinically studied and shown to be well tolerated in humans (phase I and II) in fibrosis, colorectal cancer and ovarian cancer.^[103]^ Lu et al.^[104]^ designed and synthesized several novel multifunctional pH‐sensitive amino lipids for intracellular delivery of the CRISPR/Cas9 system by modifying the amino head group. These pH‐sensitive multifunctional amino lipids, together with DNA plasmids, exhibited structure‐dependent stable nanoparticle formulations and could be used in the CRISPR/Cas9 system. Amino lipid plasmid DNA nanoparticles exhibited pH‐sensitive hemolysis with minimal hemolytic activity at pH 7.4 and increased hemolysis at acidic pH (pH = 5.5, 6.5). Amino lipids formed stable nanoparticles with high expression of Cas9 and sgRNA, and ECO‐mediated green fluorescent protein mRNA reduction of nearly 80%.

**FIGURE 7:**
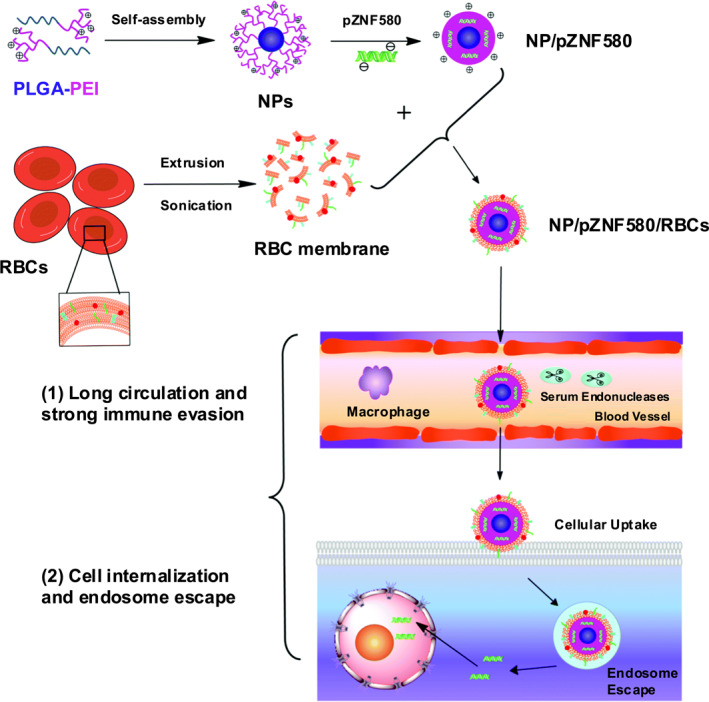
Preparation process of NP/pZNF580/RBCs and their gene delivery by crossing extracellular and intracellular barriers. Reproduced with permission.^[105]^ Copyright © 2018 Royal Society of Chemistry.

Feng et al.^[105]^ proposed an intelligent biomimetic gene delivery system based on amphiphilic PLGA‐PEI nanoparticles with a typical core‐shell structure, loaded with pZNF580 plasmid, and coated with nanoscale red blood cell (RBC) membranes through electrostatic interactions to obtain high blood compatibility and long circulation time (Figure 7). The natural cell membrane coating of NP/pZNF580/RBC ensures its immune evasion ability, especially PEI therein effectively promotes lysosomal escape, thus improving the transfection efficiency.

In recent decades, gene therapy has developed into a rapidly growing research field with many potential development and development opportunities for pre‐ and post‐clinical studies and clinical drugs, which can be applied to various fields from single‐gene diseases to common diseases, childhood leukemias to adult diseases. Among them, gene therapy will be the most important application direction for the treatment of certain genetic diseases. Using genetic engineering technology, diseases can be regulated and treated at a more basic genetic level. Rational design of gene delivery systems based on lysosomal escape mechanisms will improve the delivery efficiency of genes and provide ideas for the construction of nanocarriers.

## 5 CONCLUSIONS AND OUTLOOKS

Endo/lysosomal escape plays a crucial role in drug delivery. Traditional drug delivery methods often result in the entrapment of therapeutic cargoes within lysosomes after cellular uptake, leading to premature degradation of the drugs and ineffective delivery to the target sites, significantly reducing the bioavailability.[^106^, ^107^] In this review, we summarize various mainstream mechanisms of lysosomal escape for nanomaterials and discuss material design strategies to promote such escape. In addition, studies often involve incorporating lysosomal escape agents (CO_2_, cell‐penetrating peptides and phenylboronic acid) into nanoparticles or surface‐modifying nanoparticles to assist lysosomal escape.^[85^ ^,^ ^108^ ^,^ ^109]^

A recent review divides the evaluation of endo/lysosomal escape into two major categories, including those performed using artificial membranes or in real cells.^[47]^ For endo/lysosomal escape detection in real cells, the mainstream approach is to use fluorescent probes to detect co‐localizatioon of nanoparticles/molecules and endo/lysosomes. Typically, such strategies rely on additional chemical modifications of nanomaterials, which may lead to changes of the surface chemical properties of the materials, thereby affecting the reliability of experimental outcomes. In recent years, the vigorous development of high‐throughput analytical techniques and high‐resolution imaging techniques has provided new possibilities for advancing the evaluation of lysosomal escape of nanomaterials. (1) To address the problem that fluorescent molecular labels may change the surface properties of nanoparticles, emphasis can be placed on developing label‐free high‐resolution imaging techniques. Xu et al.^[110]^ used cryo‐soft X‐ray tomography ( cryo‐soft X‐ray tomography) technology to demonstrate a three‐dimensional distribution map of label‐free citrate‐coated silver nanoparticles in microalgae and the interaction between the nanoparticles and subcellular structures in a near‐natural manner. (2) To establish more reliable criteria for assessing lysosomal escape, it is recommended that subcellular quantitative analysis be applied to this field. For example, recent advances have made it possible to use nanosecondary ion mass spectrometry for absolute quantification at the organelle level.^[111]^ Brunet et al.^[112]^ used this technology to accurately quantify the content and spatial distribution of ^18^O‐cholesterol and ^15^N‐sphingolipids in subcellular structures. (3) High‐fidelity imaging and long‐term visualization of lysosomes are crucial for accurately detecting endo/lysosomal escape. Certain aggregation‐induced emission (AIE)‐based fluorescent trackers demonstrate extremely high quantum yields, intense fluorescence, superior resolution, reduced photobleaching, and minimal diffusion from lysosomes compared to commercial lysotracker agents.[^113^, ^114^, ^115^] These properties render AIE‐based trackers an excellent tool for real‐time monitoring of endo/lysosomal processes.

In addition, most studies evaluating the lysosomal escape functionality of materials are currently conducted at the cellular level, and few studies extend observations to in vivo models.^[116]^ The functionality of nanomedicines in biological systems is very complex, and the lack of corresponding animal experiments will seriously undermine the reliability of conclusions regarding the impact of nanomaterial lysosomal escape on the ultimate immunological or therapeutic effects. Therefore, future research is expected to not only develop smarter and more efficient lysosomal escape methods but also focus on improving the evaluation technology of nanomaterial lysosomal escape. [^110^, ^111^, ^112^] With the development of imaging technology and high‐throughput analytical methods, it is hoped that, future research will be able to more accurately understand the interactions between nanomaterials and endo/lysosomes, and develop more effective and versatile nanoparticle lysosomal escape strategies.

## CONFLICT OF INTEREST STATEMENT

The authors declare no conflicts of interest.

## Data Availability

The data that support the findings of this study are available from the corresponding author upon reasonable request.
